# The Use of Anhydrous Barium Hydroxide for Selective Alkylation of Dialkyloxy-*tert*-butyl-calix[4]arenes

**DOI:** 10.3390/molecules28031089

**Published:** 2023-01-21

**Authors:** Oleksandr A. Yesypenko, Oleksandr O. Trybrat, Vitaly I. Kalchenko

**Affiliations:** 1 Institute of Organic Chemistry NAS of Ukraine, Akademika Kuharya Str., 5, 02660 Kyiv, Ukraine; 2 Department of Chemistry and Biotechnology, Tallinn University of Technology (TalTech), Akadeemia tee, 15, 12618 Tallinn, Estonia

**Keywords:** calix[4]arenes, anhydrous barium hydroxide, selective alkylation

## Abstract

A practical method of selective alkylation of the third hydroxyl group of disubstituted *tert*-butyl-calix[4]arenes using anhydrous barium hydroxide as a base was developed in this study. The use of this method in the synthesis of inherently chiral derivatives is shown herein.

## 1. Introduction

Calixarenes are unique macrocyclic compounds that display a lipophilic cup-like structure that can be easily functionalized at different positions. They are widely used as effective and selective receptors of ions and molecules [1]. Inherently chiral calixarenes with an asymmetric placement of achiral substituents on the calixarene platform are of particular interest [2,3,4,5]. The receptors that are based on such compounds can recognize and bind enantiomers of chiral “guest” molecules and are promising for use in chiral technologies. Therefore, the development of convenient, preparative methods for the synthesis of inherently chiral calix[4]arenes is an urgent task in organic and supramolecular chemistry.

The standard method for obtaining inherently chiral derivatives of calix[4]arene consists of the sequential functionalization of the lower or upper rim of the macrocycle. The alkylation of phenolic hydroxyl groups is the most important method of macrocycle modification. The methods of monoalkylation [6,7] and distal [6,8] or proximal [9,10] alkylation of the second phenolic group are well developed and are often used. At the same time, before the publication of our work, the practical methods of the selective substitution of the third phenolic hydrogen group had not been developed.

It is known from the literature that the alkylation of calix[4]arene in anhydrous DMF in the presence of barium hydroxide as a base leads, mainly, to the tri-substituted derivatives in the *cone* conformation. Despite the fact that this reaction has been known since 1983 [11], and has been used in almost 40 works, in almost all of these cases the alkylation of tetrahydroxy-calix[4]arenes was carried out, and products with three identical alkyl groups on the lower rim were obtained. The alkylation of the third phenolic group in distally substituted calixarenes has been described in only three works [12,13,14], and inherently chiral derivatives were obtained in only one of them [13].

In all cases of alkylation using this method, a mixture of Ba(OH)_2_·8H_2_O and BaO was used instead of anhydrous barium hydroxide. When the alkylation of bromoacetic acid esters was carried out, which can be hydrolyzed under the action of barium hydroxide, only BaO was used [15,16]. However, the use of such reagents and their mixtures has a number of significant disadvantages for carrying out a selective process. It is clear that BaO was added to bind water, which is released during the interaction of the barium hydroxide with a hydroxyl group, as well as hydrate water. At the same time, an additional amount of Ba(OH)_2_ is formed, which can significantly affect the result of the alkylation. It is also known that, when it is stored, some part of the barium hydroxide goes into carbonate, and the barium oxide also goes into hydroxide and into carbonate. However, to accurately determine the content of the main substance in Ba(OH)_2_·8H_2_O and BaO is very difficult. Therefore, in the literature, we observed the different ratios of the reagents and the conditions of reaction. In terms of one hydroxyl group, from 0.3 to 1.2 equivalents Ba(OH)_2_·8H_2_O and from 0.5 to 6.6 equivalents BaO were taken. The temperature of the process varied from room temperature to 70 °C, and the reaction time varied from 0.5 h to 7 days. The yields of the tri-substituted calixarenes ranged from 20 to 80%. In addition, BaO is quite an expensive reagent.

We have noticed that the use of anhydrous barium hydroxide allows the removal of almost all of the disadvantages. This reagent is easily obtained by the dehydration of cheap Ba(OH)_2_·8H_2_O. The content of the main substance in it is determined very simply, and a small excess of anhydrous hydroxide can serve as a good water-binding agent. In this work, we have investigated the possibility of using anhydrous barium hydroxide for the selective alkylation of 1,2-dialkyloxy- and 1,3-dialkyloxy-*tert*-butyl-calix[4]arenes in order to obtain inherently chiral derivatives.

## 2. Results and Discussion

Anhydrous barium hydroxide is easily obtained by the dehydration of commercial and cheap barium hydroxide octahydrate. In order to remove the hydrate water, it was heated up to 140–150 °C under vacuum conditions to a constant weight. During storage, barium hydroxide slowly reacts with carbon dioxide and moist air and turns into barium carbonate BaCO_3_. The amount of carbonate in the anhydrous reagent is easy to determine. In order to perform this, a sample of the substance must be dissolved in warm water; the barium hydroxide quickly and completely dissolves in the water, while the carbonate remains as a precipitate. The precipitate is filtered, dried, and weighed, then the amount of barium hydroxide is calculated (see Section 3). Dry anhydrous barium hydroxide should be stored in conditions without access to moisture and carbon dioxide from the air.

It is known that carbonates of alkaline metals are used as bases in the alkylation of calixarenes and lead to di- or tetra-substituted products [6]. In order to determine whether the presence of barium carbonate will affect the alkylation processes, we investigated the interaction of dipropyloxy-calixarenes **1a** and **2a** with propyl bromide in the presence of BaCO_3_. The experiments have shown that, with a such base, alkylation does not occur either at room temperature or when it is heated to 60–70 °C. That means that barium carbonate impurities do not interfere with this reaction and will not affect the composition of the products.

Next, we studied the possibility of selective alkylation of the third hydroxyl group on 1,2-dipropyloxy [9] **1a** and 1,3-dipropyloxy [6] **2a** derivatives of *tert*-butyl-calix[4]arene in the presence of anhydrous barium hydroxide. The alkylation was carried out in dry DMF with a different ratio of reagents. The qualitative and quantitative composition of the reaction mixture was determined using ^1^H NMR spectra in a CDCl_3_ solution. The following characteristic, separately placed ^1^H signals were used for integration and comparison:

- 8.88 ppm (2H, OH) for 1,2-dipropyloxy-calix[4]arene **1a**;
- 7.87 ppm (2H, OH) for 1,3-dipropyloxy-calix[4]arene **2a**;
- 5.57 ppm (1H, OH) and 6.49 ppm (2H, Ar) for tripropyloxy-calix[4]arene **3a**;
- 6.76 ppm (8H, Ar) for tetrapropyloxy-calix[4]arene **4a**.

**Scheme 1:**
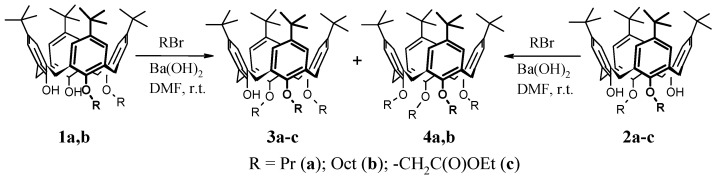
Alkylation of dialkyloxy-calix[4]arenes **1** and **2**.

The alkylation of the third hydroxyl group of calix[4]arenes **1a** and **2a** with propyl bromide proceeded very easily in 18–20 h at room temperature, and tripropyloxy-calix[4]arene **3a** was formed with a yield of 95–96% (Scheme 1). In both cases, the formation of 3–4% of the tetrasubstituted derivative **4a** was also recorded. The alkylation of 1,2-dioctyloxy- **1b** and 1,3-dioctyloxy- **2b** calix[4]arenes with octyl bromide was similar, but slightly more (up to 5–6%) of the corresponding tetrasubstituted product of **4b** was formed.

In order to check whether complete substitution to tetrapropyloxy-calix[4]arene **4a** can occur under these reaction conditions, we investigated the alkylation of tripropyloxy-calixarene **3a**. It was found that further alkylation of the compound **3a** at room temperature practically does not occur. Even in the presence of a 1.5-fold excess of Ba(OH)_2_ after long-term mixing, no more than 9% of tetrapropiloxy-calix[4]arene **4a** was formed. When it is heated, the amount of product **4a** increases, but does not exceed 30%. Tetrasubstituted calixarene **4a** can be separated by crystallization from DMF (**4a** is precipitated) or by column chromatography.

We have also shown that there is no hydrolysis of ester groups in these conditions. Alkylation with the ethyl ester of the bromoacetic acid of calixarene **2c** with two distally placed ester groups led to the formation of corresponding trisubstituted product **3c**, which was isolated in 95% yield after column chromatography. Therefore, anhydrous barium hydroxide can be used for alkylation with esters of bromoacetic acid.

Thus, the anhydrous barium hydroxide is a convenient reagent for the selective alkylation of calix[4]arenes. The amount of base should be slightly larger than the equimolar and sufficient to bind the water. An excess of barium hydroxide of 20–30% is the best option. The reaction should be carried out at room temperature (20–25 °C) while stirring the mixture for at least 20 h. However, before adding an alkylating reagent, the mixture of calixarene with barium hydroxide should be mixed for 30–40 min at 40–50 °C for complete salt formation (except for calixarenes containing an ester group).

**Scheme 2:**
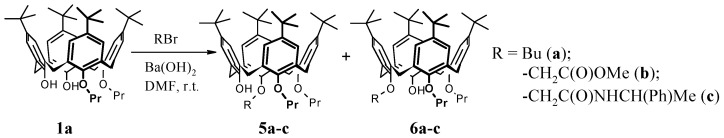
Asymmetric alkylation of 1,2-dipropyloxy-calix[4]arene **1a**.

The described alkylation method allows us to easily obtain inherently chiral derivatives. The corresponding racemic mixture of trialkyloxy-calix[4]arenes **5a** + **6a** or **5b** + **6b** with a total yield of 94–96% was formed upon the alkylation of 1,2-dipropyloxy-calix[4]arene **1a** with butyl bromide or methyl ester of bromoacetic acid (Scheme 2).

**Figure 1:**
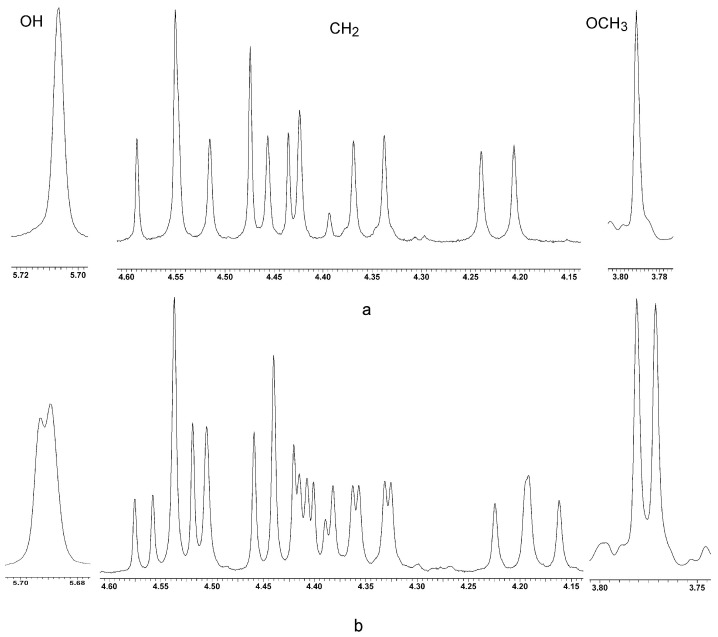
Representative parts of the ^1^H NMR spectra of the enantiomeric pairs **5b** + **6b** before (**a**) and after (**b**) the addition of the Pirkle reagent in CDCl_3_ at 20 °C (400 MHz).

The presence of two enantiomer structures in a 1:1 ratio was clearly visible in the ^1^H NMR spectra with the addition of the Pirkle reagent [(*S*)-2,2,2-thrifluoro-1-(9-anthryl)ethanol]. Splitting of the ^1^H signals of the methyl and hydroxyl groups, as well as bridging protons, was also observed (Figure 1).

The alkylation of compound **1a** with optically pure chiral N-(1-phenylethyl)amide of bromoacetic acid led to the formation of a pair of diastereomers, **5c** + **6c,** with a *de* of 33% and a total yield of 96%. The stereoisomers were separated using column chromatography or crystallization [17].

**Scheme 3:**
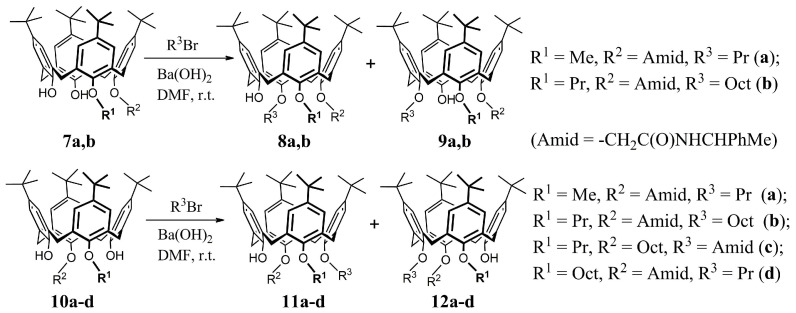
Alkylation of dialkyloxy-calix[4]arenes **7** and **10**.

We also used this method to obtain inherently chiral calixarenes with three different substituents on the lower rim. For this purpose, we selectively alkylated the calixarene derivatives with two different alkyl groups in the proximal **7a,b** or distal **10a**–**d** position [18,19] (Scheme 3). In the case of the alkylation of the proximally substituted derivative **7b** with octyl bromide, the reaction took place at 100 °C. Depending on the nature of the substituents in the starting compounds and the order of their placement on the macrocyclic platform, the ratio of iteromeric products **8**/**9** and **11**/**12** was observed from 1:1 to 9:1, with a total yield of 92–98%. The side tetrasubstituted product was formed in an amount of up to 3–4% and was easily removed by column chromatography.

All of the trisubstituted compounds that were obtained with this method had the *cone* conformation, which was confirmed by NMR spectra. Thus, in the ^1^H NMR spectra, the signals of the axial and equatorial protons of the methylene bridges appeared as four pairs of doublets with the spin–spin coupling constant 12.5–13.9 Hz, and the distance between the signals of the Ar-CH-*eq* and Ar-CH-*ax* protons was greater than 0.5 ppm [20,21].

## 3. Materials and Methods

The NMR spectra were registered on a Varian VXR-400 spectrometer operating a 400 MHz (^1^H) using TMS as a reference. The melting points were measured on a Boёtius heating block and are uncorrected. The reactions were carried out in anhydrous solvents. DMF was purified by the azeotropic drying method with benzene. The column chromatography was performed on silica gel that was purchased from Acros Organics (0.040–0.063 mm, pore diameter 6 nm).

Dialkyloxy-calix[4]arenes were obtained according to method [9] for **1a** and **1b**, [6] for **2a** and **2b**, and [15] for **2c**.

### 3.1. Preparation of Anhydrous Barium Hydroxide

The flask with commercial barium hydroxide octahydrate was heated at 140–150 °C in a vacuum (1–5 mm Hg) until the complete removal of hydrated water (to constant weight). The flask was required to be periodically stirred so that the substance remained in the form of a powder.

### 3.2. Determination of the Content of the Main Substance in Anhydrous Barium Hydroxide

A sample of barium hydroxide with a precisely determined mass *m* (about 1 g was taken) was dissolved in warm (60–70 °C) boiled (to remove soluble CO_2_) water. The solution was protected from access to CO_2_ from the air. The precipitate of water-insoluble barium carbonate was filtered off, dried, and accurately weighed *m[BaCO_3_]*. The mass of the main substance *m[Ba(OH)_2_]* was calculated according to the following formula:

*m[Ba(OH)_2_] = m − m[BaCO_3_].*

### 3.3. General Procedure for the Selective Alkylation

A mixture of dialkyloxy-calix[4]arene **1**, **2**, **7,** or **10** (0.50 mmol) and anhydrous Ba(OH)_2_ (0.15 g, 0.65 mmol) in dry DMF (5–10 mL) was stirred at 40–50 °C for 30–40 min. After cooling to room temperature, the suitable alkylating reagent was added [1.50–2.50 mmol of propyl bromine, octyl bromine, or esters of bromoacetic acid; 10.83 mmol (0.2 g) of N-(1-phenylethyl)bromoacetamide]. The mixture was stirred at 20–25 °C for 24 h. After the end of the reaction, water (10 mL) and 30% HCl (1 mL) were added to the reaction mixture and the products were extracted with CHCl_3_ (3 × 5 mL). The organic phase was washed with water (5 mL), washed with brine (5 mL), and dried over Na_2_SO_4_. After the removal of the solvent, the corresponding compounds, or mixtures of compounds, were isolated.

*25,26,27-Tripropyloxy-tert-butyl-calix[4]arene ***3a** had a yield of 95% from **1a** and 96% from **2a**. The spectral characteristics completely coincide with the literature [20].

*25,26,27-Trioctyloxy-tert-butyl-calix[4]arene ***3b** had a yield of 96% from **1b** and 94% from **2b**. The spectral characteristics completely coincide with the literature [22].

*25,26,27-Tri(ethyloxycarbonylmethyloxy)-tert-butyl-calix[4]arene* ***3c*** had a yield of 95% from **2c**. The spectral characteristics completely coincide with the literature [15].

*Dipropyloxy-buthyloxy-tert-butyl-calix[4]arenes***5a** + **6a** had a total yield of 94%, m.p. 99–100 °C (MeCN). ^1^H NMR (CDCl_3_), δ: 0.81 (s, 18H, *t*-Bu), 0.94 (t, 3H, Me^Pr^, ^3^*J_HH_* = 7.3 Hz), 0.99 (t, 3H, Me^Pr^, ^3^*J_HH_* = 7.3 Hz), 1.09 (t, 3H, Me^Bu^, ^3^*J_HH_* = 7.3 Hz), 1.32 (s, 9H, *t-*Bu), 1.33 (s, 9H, *t*-Bu), 1.46–1.68 (m, 2H, -C*H_2_*-), 1.75–2.00 (m, 4H, -C*H_2_*-), 2.25-2.39 (m, 2H, -C*H_2_*-), 3.16 (d, 2H, Ar-C*H_2_*-eq, ^2^*J_HH_* = 12.7 Hz), 3.22 (d, 2H, Ar-C*H_2_*-eq, ^2^*J_HH_* = 13.0 Hz), 3.74 (t, 2H, OCH_2_, ^3^*J_HH_* = 6.7 Hz), 3.77 (t, 2H, OCH_2_, ^3^*J_HH_* = 6.7 Hz), 3.83 (t, 2H, OCH_2_, ^3^*J_HH_* = 8.4 Hz), 4.31 (d, 1H, Ar-C*H_2_*-ax, ^2^*J_HH_* = 13.0 Hz), 4.32 (d, 1H, Ar-C*H_2_*-ax, ^2^*J_HH_* = 13.0 Hz), 4.35 (d, 1H, Ar-C*H_2_*-ax, ^2^*J_HH_* = 12.7 Hz), 4.36 (d, 1H, Ar-C*H_2_*-ax, ^2^*J_HH_* = 12.7 Hz), 5.57 (s, 1H, O*H*), 6.50 (s, 4H, Ar*H*), 7.04 (s, 2H, Ar*H*), 7.13 (s, 2H, Ar*H*). The calc. % was C 82.18; H 9.71. C_54_H_76_O_4_. The found % was C 82.29; H 9.79.

*Dipropyloxy-(methyloxycarbonylmethyloxy)-tert-butyl-calix[4]arenes***5b** + **6b** had a total yield of 95%, m.p. 87–89 °C (MeCN). ^1^H NMR (CDCl_3_), δ: 0.77 (s, 9H, *t*-Bu), 0.89 (s, 9H, *t*-Bu), 0.95 (t, 3H, Me^Pr^, ^3^*J_HH_* = 7.3 Hz), 1.09 (t, 3H, Me^Pr^, ^3^*J_HH_* = 7.3 Hz), 1.31 (s, 9H, *t-*Bu), 1.32 (s, 9H, *t*-Bu), 1.82–2.06 (m, 2H, -C*H_2_*-), 2.14–2.37 (m, 2H, -C*H_2_*-), 3.17 (d, 1H, Ar-C*H_2_*-eq, ^2^*J_HH_* = 12.5 Hz), 3.19 (d, 1H, Ar-C*H_2_*-eq, ^2^*J_HH_* = 12.9 Hz), 3.22 (d, 1H, Ar-C*H_2_*-eq, ^2^*J_HH_* = 13.4 Hz), 3.27 (d, 1H, Ar-C*H_2_*-eq, ^2^*J_HH_* = 13.4 Hz), 3.71-3.90 (m, 4H, OCH_2_), 3.79 (s, 3H, MeO), 4.22 (d, 1H, Ar-C*H_2_*-ax, ^2^*J_HH_* = 13.4 Hz), 4.35 (d, 1H, Ar-C*H_2_*-ax, ^2^*J_HH_* = 12.9 Hz), 4.44 (d, 1H, Ar-C*H_2_*-ax, ^2^*J_HH_* = 12.5 Hz), 4.45 (d, 1H, OC*H_2_*, ^2^*J_HH_* = 15.6 Hz), 4.53 (d, 1H, Ar-C*H_2_*-ax, ^2^*J_HH_* = 13.4 Hz), 4.57 (d, 1H, OC*H_2_*, ^2^*J_HH_* = 15.6 Hz), 5.71 (s, 1H, O*H*), 6.45 (s, 2H, Ar*H*), 6.61 (s, 1H, Ar*H*), 6.64 (s, 1H, Ar*H*), 7.01 (s, 1H, Ar*H*), 7.06 (s, 1H, Ar*H*), 7.10 (s, 1H, Ar*H*), 7.12 (s, 1H, Ar*H*). The calc. % was C 79.06; H 9.01. C_53_H_72_O_6_. The found % was C 80.02; H 9.13.

*Dipropyloxy-N-(α-phenylethyl)aminocarbonylmethyloxy-tert-butyl-calix[4]arenes***5c** + **6c** had a total yield of 94% (*de* of 33%) [17]. After column chromatography (hexane-ethyl acetate, 6:1), the following compounds were isolated: 

The first fraction: Calix[4]arene **6c** had a yield of 41%, m.p. 106–107 °C (MeCN). ^1^H NMR (CDCl_3_), δ: 0.58 (t, 3H, Me^Pr^, ^3^*J_HH_* = 7.8 Hz), 0.86 (s, 9H, *t*-Bu), 0.91 (s, 9H, *t*-Bu), 0.98 (t, 3H, Me^Pr^, ^3^*J_HH_* = 7.5 Hz), 1.27 (s, 9H, *t*-Bu), 1.32 (s, 9H, *t*-Bu), 1.55 (d, 3H, Me^Amid^, ^3^*J_HH_* = 7.2 Hz), 1.73–1.91 (m, 4H, -C*H_2_*-), 3.17 (d, 2H, Ar-C*H_2_*-eq, ^2^*J_HH_* = 12.4 Hz), 3.22 (d, 1H, Ar-C*H_2_*-eq, ^2^*J_HH_* = 12.9 Hz), 3.37 (d, 1H, Ar-C*H_2_*-eq, ^2^*J_HH_* = 13.4 Hz), 3.71–3.91 (m, 4H, OC*H_2_*), 4.08 (d, 1H, C*H_2_*CO, ^2^*J_HH_* = 16.0 Hz), 4.16 (d, 1H, Ar-C*H_2_*-ax, ^2^*J_HH_* = 13.4 Hz), 4.31 (d, 1H, Ar-C*H_2_*-ax, ^2^*J_HH_* = 12.9 Hz), 4.33 (d, 2H, Ar-C*H_2_*-ax, ^2^*J_HH_* = 12.4 Hz), 4.73 (d, 1H, C*H_2_*CO, ^2^*J_HH_* = 16.0 Hz), 5.23–5.34 (m, 1H, C*H*), 5.75 (s, 1H, O*H*), 6.59 (s, 1H, Ar*H*), 6.61 (s, 1H, Ar*H*), 6.67 (s, 1H, Ar*H*), 6.71 (s, 1H, Ar*H*), 7.08 (s, 2H, Ar*H*), 7.09 (s, 1H, Ar*H*), 7.11 (s, 1H, Ar*H*), 7.26–7.31 (m, 1H, Ph*H*), 7.33–7.40 (m, 2H, Ph*H*), 7.49 (d, 2H, Ph*H*), 8.68 (d, 1H, N*H*, ^3^*J_HH_* = 7.5 Hz). The calc. % was C 79.44; H 8.60; N 3.29. C_60_H_79_NO_5_ + CH_3_CN. The found % was C 79.16; H 8.81; N 3.66.

The second fraction: Calix[4]arene **5c** had a yield of 69%, m.p. 104 °C (MeCN). ^1^H NMR (CDCl_3_), δ: 0.84 (s, 9H, *t*-Bu), 0.87 (s, 9H, *t*-Bu), 0.94 (t, 3H, Me^Pr^, ^3^*J_HH_* = 7.6 Hz), 1.09 (t, 3H, Me^Pr^, ^3^*J_HH_* = 7.6 Hz), 1.30 (s, 9H, *t*-Bu), 1.32 (s, 9H, *t*-Bu), 1.64 (d, 3H, Me^Amid^, ^3^*J_HH_* = 7.1 Hz), 1.88–2.02 (m, 2H, -C*H_2_*-), 2.05–2.17 (m, 2H, -C*H_2_*-), 3.04 (d, 1H, Ar-C*H_2_*-eq, ^2^*J_HH_* = 12.9 Hz), 3.19 (d, 1H, Ar-C*H_2_*-eq, ^2^*J_HH_* = 12.5 Hz), 3.21 (d, 1H, Ar-C*H_2_*-eq, ^2^*J_HH_* = 12.5 Hz), 3.35 (d, 1H, Ar-C*H_2_*-eq, ^2^*J_HH_* = 13.4 Hz), 3.72–3.80 (m, 1H, OC*H_2_*), 3.84–3.94 (m, 2H, OC*H_2_*), 3.98–4.06 (m, 1H, OC*H_2_*), 4.01 (d, 1H, C*H_2_*CO, ^2^*J_HH_* = 15.4 Hz), 4.09 (d, 1H, Ar-C*H_2_*-ax, ^2^*J_HH_* = 12.9 Hz), 4.17 (d, 1H, Ar-C*H_2_*-ax, ^2^*J_HH_* = 13.4 Hz), 4.34 (d, 1H, Ar-C*H_2_*-ax, ^2^*J_HH_* = 12.4 Hz), 4.39 (d, 1H, Ar-C*H_2_*-ax, ^2^*J_HH_* = 12.4 Hz), 4.69 (d, 1H, C*H_2_*CO, ^2^*J_HH_* = 15.4 Hz), 5.24–5.33 (m, 1H, C*H*), 5.66 (s, 1H, O*H*), 6.55 (s, 1H, Ar*H*), 6.58 (s, 1H, Ar*H*), 6.62 (s, 1H, Ar*H*), 6.69 (s, 1H, Ar*H*), 7.00–7.08 (m, 5H, Ph*H*), 7.11 (s, 1H, Ar*H*), 7.12 (s, 1H, Ar*H*), 7.30–7.33 (m, 2H, Ar*H*), 8.79 (d, 1H, N*H*, ^3^*J_HH_* = 8.3 Hz). The calc. % was C 80.58; H 8.90; N 1.57. C_60_H_79_NO_5_. The found % was C 80.92; H 8.83; N 1.36.

*Methyloxy-propyloxy-N-(α-phenylethyl)aminocarbonylmethyloxy-tert-butyl-calix[4]arenes***8a** + **9a** had a total yield of 94% [18]. Product **8a** was formed regioselectively with a trace amount of the isomer **9a**. Calixarene **8a** was isolated after crystallization from MeCN with a yield of 63%. M.p. 206 °C (MeCN). ^1^H NMR (CDCl_3_), δ: 0.80 (s, 9H, *t*-Bu), 0.85 (s, 9H, *t*-Bu), 1.12 (t, 3H, Me^Pr^, ^3^*J_HH_* = 7.5 Hz), 1.34–1.41 (s, 18H, *t*-Bu), 1.67 (d, 3H, Me^Amid^, ^3^*J_HH_* = 7.2 Hz), 1.96 (m, 2H, -C*H_2_-*), 3.14 (d, 1H, Ar-C*H_2_*-eq, ^2^*J_HH_* = 13.1 Hz), 3.24 (d, 2H, Ar-C*H_2_*-eq), 3.38 (d, 1H, Ar-C*H_2_*-eq, ^2^*J_HH_* = 13.4 Hz), 3.80–3.92 (m, 2H, OC*H_2_*), 3.98 (s, 3H, OMe), 4.11–4.35 (m, 4H, Ar-C*H_2_*-ax + 1H, OC*H_2_*), 4.63 (d, 1H, OC*H_2_*, ^2^*J_HH_* = Hz), 5.30 (m, 1H, C*H*), 5.47 (s, 1H, O*H*), 6.51–6.62 (m, 4H, Ar*H*), 7.09–7.39 (m, 9H, Ar*H* + Ph*H*), 8.88 (d, 1H, N*H*, ^3^*J_HH_* = 8.7 Hz). The calc. % was C 80.42; H 8.73; N 1.62. C_58_H_75_NO_5_. The found % was C 79.98, H 8.36, N 1.68.

*Propyloxy-octyloxy-N-(α-phenylethyl)aminocarbonylmethyloxy-tert-butyl-calix[4]arenes***8b** +**9b** had a total yield of 96% (the ratio of iteromers is 9:1) [19]. After column chromatography (hexane-ethyl acetate, 5:1), the following compounds were isolated:

The first fraction: Calix[4]arene **8b** had a yield of 58%, m.p. 77–78 °C (MeCN). ^1^H NMR (CDCl_3_), δ: 0.75 (s, 9H, *t*-Bu), 0.85 (s, 9H, *t*-Bu), 0.86 (t, 3H, Me^Oct^, ^3^*J_HH_* = 6.9 Hz), 0.94 (t, 3H, Me^Pr^, ^3^*J_HH_* = 7.4 Hz), 1.06–1.32 (m, 12H, -C*H_2_*-^Oct^), 1.34 (s, 18H, *t*-Bu), 1.79–1.87 (m, 2H, -C*H_2_*-^Pr^), 1.83 (d, 3H, Me^Amid^, ^3^*J_HH_* = 7.1 Hz), 3.17 (d, 1H, Ar-C*H_2_*-eq, ^2^*J_HH_* = 12.3 Hz), 3.19 (d, 1H, Ar-C*H_2_*-eq, ^2^*J_HH_* = 13.2 Hz), 3.24 (d, 1H, Ar-C*H_2_*-eq, ^2^*J_HH_* = 12.6 Hz), 3.28–3.38 (m, 2H, OC*H_2_*), 3.32 (d, 1H, Ar-C*H_2_*-eq, ^2^*J_HH_* = 13.2 Hz), 3.72–3.89 (m, 2H, OC*H_2_*), 4.22 (d, 1H, Ar-*CH_2_-*ax, ^2^*J_HH_* = 12.6 Hz), 4.23 (d, 1H, Ar-C*H_2_*-ax, ^2^*J_HH_* = 13.2 Hz), 4.24 (d, 1H, Ar-C*H_2_*-ax, ^2^*J_HH_* = 12.3 Hz), 4.27 (d, 1H, Ar-C*H_2_*-ax, ^2^*J_HH_* = 13.2 Hz), 4.37 (d, 1H, C*H_2_*CO, ^2^*J_HH_* = 14.3 Hz), 4.45 (d, 1H, C*H_2_*CO, ^2^*J_HH_* = 14.3 Hz), 5.34–5.40 (m, 1H, C*H*), 6.39 (s, 1H, O*H*), 6.42 (s, 1H, Ar*H*), 6.48 (s, 1H, Ar*H*), 6.57 (s, 1H, Ar*H*), 6.66 (s, 1H, Ar*H*), 7.07 (s, 1H, Ar*H*), 7.10 (s, 1H, Ar*H*), 7.15 (s, 1H, Ar*H*), 7.17 (s, 1H, Ar*H*), 7.23 (t, 1H, PhH, ^3^*J_HH_* = 7.4 Hz), 7.32 (t, 2H, PhH, ^3^*J_HH_* = 7.4 Hz), 7.60 (d, 2H, PhH, ^3^*J_HH_* = 7.4 Hz), 8.63 (s, 1H, NH). The calc. % was C 80.95, H 9.30, N 1.45. C_65_H_89_NO_5_. The found % was C 81.17, H 9.23, N 1.26.

The second fraction: Calix[4]arene **9b** had a yield of 13%, m.p. 175–178 °C (MeCN). ^1^H NMR (CDCl_3_), δ: 0.82 (s, 9H, *t*-Bu), 0.85 (s, 9H, *t*-Bu), 0.86 (t, 3H, Me^Oct^, ^3^*J_HH_* = 6.6 Hz), 0.94 (t, 3H, Me^Pr^, ^3^*J_HH_* = 7.4 Hz), 1.22–1.31 (m, 10H, -C*H_2_*-^Oct^), 1.31 (s, 9H, *t*-Bu), 1.33 (s, 9H, *t*-Bu), 1.65 (d, 3H, Me^Amid^, ^3^*J_HH_* = 6.9 Hz), 1.86–2.00 (m, 2H, -C*H_2_*-^Pr^), 2.08–2.16 (m, 2H, -C*H_2_*-^Pr^), 3.03 (d, 1H, Ar-C*H_2_*-eq, ^2^*J_HH_* = 13.2 Hz), 3.19 (d, 1H, Ar-C*H_2_*-eq, ^2^*J_HH_* = 12.3 Hz), 3.21 (d, 1H, Ar-C*H_2_*-eq, ^2^*J_HH_* = 12.3 Hz), 3.35 (d, 1H, Ar-C*H_2_*-eq, ^2^*J_HH_* = 13.4 Hz), 3.76–3.82 (m, 1H, OC*H_2_*), 3.87–3.93 (m, 2H, OC*H_2_*), 4.00–4.06 (m, 1H, OC*H_2_*), 4.02 (d, 1H, C*H_2_*CO, ^2^*J_HH_* = 15.4 Hz), 4.07 (d, 1H, Ar-C*H_2_*-ax, ^2^*J_HH_* = 13.2 Hz), 4.18 (d, 1H, Ar-C*H_2_*-ax, ^2^*J_HH_* = 13.4 Hz), 4.35 (d, 1H, Ar-C*H_2_*-ax, ^2^*J_HH_* = 12.3 Hz), 4.39 (d, 1H, Ar-C*H_2_*-ax, ^2^*J_HH_* = 12.3 Hz), 4.69 (d, 1H, C*H_2_*CO, ^2^*J_HH_* = 15.4 Hz), 5.25–5.31 (m, 1H, C*H*), 5.45 (s, 1H, O*H*), 6.52 (s, 1H, Ar*H*), 6.55 (s, 1H, Ar*H*), 6.59 (s, 1H, Ar*H*), 6.65 (s, 1H, Ar*H*), 7.01 (s, 1H, Ar*H*), 7.04 (t, 2H, Ph*H*, ^3^*J_HH_* = 7.0 Hz), 7.05 (t, 1H, Ph*H*, ^3^*J_HH_* = 7.0 Hz), 7.06 (s, 1H, Ar*H*), 7.11 (s, 1H, Ar*H*), 7.13 (s, 1H, Ar*H*), 7.32 (d, 2H, Ph*H*, ^3^*J_HH_* = 7.4 Hz), 8.78 (d, 1H, N*H*, ^3^*J_HH_* = 8.2 Hz). The calc. % was C 80.95, H 9.30, N 1.45. C_65_H_89_NO_5_. The found % was C 79.37, H 8.93, N 1.52.

*Methyloxy-propyloxy-N-(α-phenylethyl)aminocarbonylmethyloxy-tert-butyl-calix[4]arenes***11a** + **12a** had a total yield of 92% (the ratio of stereomers was 1:1) [18]. After column chromatography (hexane-ethyl acetate, 6:1), the following compounds were isolated:

The first fraction: Calix[4]arene **11a** had a yield of 38%; m.p. 204–205 °C (MeCN). ^1^H NMR (CDCl_3_), δ: 0.62 (t, 3H, Me^Pr^, ^3^*J_HH_* = 7.5 Hz), 0.88 (s, 9H, *t*-Bu), 0.97 (s, 9H, *t*-Bu), 1.22 (s, 9H, *t*-Bu), 1.30 (s, 9H, *t*-Bu), 1.56 (d, 3H, Me^Amid^, ^2^*J_HH_* = 3.7 Hz), 1.70 (m, 2H, -C*H_2_*-), 3.16–3.25 (d, 3H, Ar-C*H_2_*-eq), 3.41 (d, 1H, Ar-C*H_2_*-eq, ^2^*J_HH_* = 13.7 Hz), 3.60–3.80 (m, 2H, OC*H_2_*), 3.68 (s, 3H, OMe), 4.09 (d, 1H, C*H_2_*CO, ^2^*J_HH_* = 14.9 Hz), 4.10 (d, 1H, Ar-C*H_2_*-ax, ^2^*J_HH_* = 12.5 Hz), 4.23 (d, 1H, Ar-C*H_2_*-ax, ^2^*J_HH_* = 12.5 Hz), 4.31 (d, 1H, Ar-C*H_2_*-ax, ^2^*J_HH_* = 12.5 Hz), 4.34 (d, 1H, Ar-C*H_2_*-ax, ^2^*J_HH_* = 13.1 Hz), 4.77 (d, 1H, C*H_2_*CO, ^2^*J_HH_* = 14.9 Hz), 5.33 (m, 1H, C*H*), 6.65 (s, 1H, O*H*), 6.75 (s, 1H, Ar*H*), 6.81 (s, 2H, Ar*H*), 7.00 (s, 1H, Ar*H*), 7.05 (s, 2H, Ph*H*), 7.11 (s, 1H, Ar*H*), 7.32 (s, 1H, Ar*H*), 7.40 (m, 3H, Ph*H*), 7.56 (m, 2H, Ph*H*), 8.96 (d, 1H, N*H*, ^3^*J_HH_* = 8.1 Hz). The calc. % was C 80.42; H 8.73; N 1.62. C_58_H_75_NO_5_. The found, % was C 79.98, H 8.24, N 1.89.

The second fraction: Calix[4]arene **12a** had a yield of 22%, m.p. 155–156 °C (MeOH-H_2_O). ^1^H NMR (CDCl_3_), δ: 0.87 (s, 9H, *t*-Bu), 0.97 (s, 9H, *t*-Bu), 1.01 (t, 3H, Me^Pr^, ^3^*J_HH_* = 7.5 Hz), 1.25 (s, 9H, *t*-Bu), 1.30 (s, 9H, *t*-Bu), 1.69 (d, 3H, Me^Amid^, ^3^*J_HH_* = 6.8 Hz), 2.09 (m, 2H, -C*H_2_*-), 3.08 (d, 1H, Ar-C*H_2_*-eq, ^2^*J_HH_* = 12.8 Hz), 3.23 (d, 1H, Ar-C*H_2_*-eq, ^2^*J_HH_* = 12.5 Hz), 3.25 (d, 1H, Ar-C*H_2_*-eq, ^2^*J_HH_* = 12.5 Hz), 3.38 (d, 1H, Ar-C*H_2_*-eq, ^2^*J_HH_* = 13.7 Hz), 3.83-3.96 (m, 2H, OC*H_2_*), 3.91 (s, 3H, OMe), 4.03 (d, 1H, C*H_2_*CO, ^2^*J_HH_* = 15.3 Hz), 4.05 (d, 1H, Ar-C*H_2_*-ax, ^2^*J_HH_* = 13.4 Hz, Ar-C*H_2_*-Ar), 4.14 (1H, d, ^2^*J_HH_* = 12.8 Hz, Ar-C*H_2_*-Ar), 4.35 (d, 1H, Ar-C*H_2_*-ax, ^2^*J_HH_* = 12.5 Hz), 4.37 (d, 1H, Ar-C*H_2_*-ax, ^2^*J_HH_* = 12.5 Hz), 4.75 (d, 1H, C*H_2_*CO, ^2^*J_HH_* = 15.3 Hz), 5.29 (m, 1H, C*H*), 6.60 (s, 1H, Ar*H*), 6.66 (s, 1H, Ar*H*), 6.75 (s, 1H, Ar*H* + 1H, O*H*), 6.85 (s, 1H, Ar*H*), 7.01–7.06 (m, 3H, Ph*H*), 7.1 (m, 4H, Ar*H*), 7.36 (d, 2H, Ph*H*), 9.03 (d, 1H, N*H*, ^3^*J_HH_* = 8.1 Hz). The calc. % was C 80.42; H 8.73; N 1.62. C_58_H_75_NO_5_. The found % was C 80.11, H 8.27, N 1.73.

*Propyloxy-octyloxy-N-(α-phenylethyl)aminocarbonylmethyloxy-tert-butyl-calix[4]arenes***11b** + **12b** had a total yield of 98% (*de* of 30%) [19]. After column chromatography (hexane-ethyl acetate, 10:1), the following compounds were isolated:

The first fraction: Calix[4]arene **12b** had a yield of 26%, m.p. 74–76 °C (MeCN). ^1^H NMR (CDCl_3_), δ: 0.85 (s, 9H, *t*-Bu), 0.88 (t, 3H, Me^Oct^, 0.89 (s, 9H, *t*-Bu), 0.98 (t, 3H, Me^Pr^, ^3^*J_HH_* = 7.4 Hz), 1.06–1.24 (m, 10H, -C*H_2_*-^Oct^), 1.27 (s, 9H, *t*-Bu), 1.31 (s, 9H, *t*-Bu), 1.54 (d, 3H, Me^Amid^, ^3^*J_HH_* = 6.9 Hz), 1.71–1.81 (m, 2H, -C*H_2_*-^Oct^), 1.82-1.89 (m, 2H, -C*H_2_*-^Pr^), 3.17 (d, 1H, Ar-C*H_2_*-eq, ^2^*J_HH_* = 12.6 Hz), 3.18 (d, 1H, Ar-C*H_2_*-eq, ^2^*J_HH_* = 12.6 Hz), 3.22 (d, 1H, Ar-C*H_2_*-eq, ^2^*J_HH_* = 12.9 Hz), 3.37 (d, 1H, Ar-C*H_2_*-eq, ^2^*J_HH_* = 13.4 Hz), 3.74–3.80 (m, 1H, OC*H_2_*), 3.81–3.89 (m, 2H, OC*H_2_*), 3.90–3.98 (m, 1H, OC*H_2_*), 4.10 (d, 1H, C*H_2_*CO, ^2^*J_HH_* = 15.4 Hz), 4.19 (d, 1H, Ar-C*H_2_*-ax, ^2^*J_HH_* = 13.4 Hz), 4.31 (d, 1H, Ar-C*H_2_*-ax, ^2^*J_HH_* = 12.6 Hz), 4.34 (d, 1H, Ar-C*H_2_*-ax, ^2^*J_HH_* = 12.9 Hz), 4.34 (d, 1H, Ar-C*H_2_*-ax, ^2^*J_HH_* = 12.6 Hz), 4.74 (d, 1H, C*H_2_*CO, ^2^*J_HH_* = 15.4 Hz), 5.25–5.32 (m, 1H, C*H*), 5.87 (s, 1H, O*H*), 6.57 (s, 1H, Ar*H*), 6.59 (s, 1H, Ar*H*), 6.65 (s, 1H, Ar*H*), 6.69 (s, 1H, Ar*H*), 7.05 (s, 1H, Ar*H*), 7.06 (s, 1H, Ar*H*), 7.07 (s, 1H, Ar*H*), 7,10 (s, 1H, Ar*H*), 7.28 (t, 1H, Ph*H*, ^3^*J_HH_* = 7.1 Hz), 7.36 (t, 2H, Ph*H*, ^3^*J_HH_* = 7.4 Hz), 7.49 (d, 2H, Ph*H*, ^3^*J_HH_* = 7.1 Hz), 8.69 (d, 1H, N*H*, ^3^*J_HH_* = 8.3 Hz). The calc. % was C 80.95, H 9.30, N 1.45. C_65_H_89_NO_5_. The found % was C 81.17, H 8.63, N 1.32. 

The second fraction: Calix[4]arene **11b** had a yield of 48%, m.p. 171–174 °C (MeCN). ^1^H NMR (CDCl_3_), δ: 0.84 (s, 9H, *t*-Bu), 0.87 (s, 9H, *t*-Bu), 0.89 (t, 3H, Me^Oct^, ^3^*J_HH_* = 6.7 Hz), 1.09 (t, 3H, Me^Pr^, ^3^*J_HH_* = 7.4 Hz), 1.21–1.37 (m, 10H, -C*H_2_*-^Oct^), 1.30 (s, 9H, *t*-Bu), 1.33 (s, 9H, *t*-Bu), 1.65 (d, 3H, Me^Amid^, ^3^*J_HH_* = 7.0 Hz), 1.88-2.00 (m, 2H, -C*H_2_*-^Oct^), 2.00–2.10 (m, 2H, -C*H_2_*-^Pr^), 3.03 (d, 1H, Ar-C*H_2_*-eq, ^2^*J_HH_* = 13.2 Hz), 3.19 (d, 1H, Ar-C*H_2_*-eq, ^2^*J_HH_* = 12.5 Hz), 3.21 (d, 1H, Ar-C*H_2_*-eq, ^2^*J_HH_* = 12.5 Hz), 3.35 (d, 1H, Ar-C*H_2_*-eq, ^2^*J_HH_* = 13.5 Hz), 3.72–3.80 (m, 1H, OC*H_2_*), 3.88–3.95 (m, 1H, OC*H_2_*), 3.97-4.05 (m, 1H, OC*H_2_*), 4.02 (d, 1H, C*H_2_*CO, ^2^*J_HH_* = 15.5 Hz), 4.06-4.11 (m, 1H, OC*H_2_*), 4.09 (d, 1H, Ar-C*H_2_*-ax, ^2^*J_HH_* = 13.2 Hz), 4.18 (d, 1H, Ar-C*H_2_*-ax, ^2^*J_HH_* = 13.5 Hz), 4.35 (d, 1H, Ar-C*H_2_*-ax, ^2^*J_HH_* = 12.5 Hz), 4.40 (d, 1H, Ar-C*H_2_*-ax, ^2^*J_HH_* = 12.5 Hz), 4.69 (d, 1H, C*H_2_*CO, ^2^*J_HH_* = 15.5 Hz), 5.21–5.29 (m, 1H, C*H*), 5.61 (s, 1H, O*H*), 6.54 (s, 1H, Ar*H*), 6.57 (s, 1H, Ar*H*), 6.60 (s, 1H, Ar*H*), 6.67 (s, 1H, *ArH*), 7.02 (s, 1H, Ar*H*), 7.05–7.08 (m, 3H, Ph*H* + 1H, Ar*H*), 7.11 (s, 1H, Ar*H*), 7.12 (s, 1H, Ar*H*), 7.31 (d, 2H, Ph*H*, ^3^*J_HH_* = 7.4 Hz), 8.82 (d, 1H, N*H*, ^3^*J_HH_* = 8.1 Hz). The calc. % was C 80.95, H 9.30, N 1.45. C_65_H_89_NO_5_. The found % was C 80.37, H 9.19, N 1.12.

*Propyloxy-octyloxy-N-(α-phenylethyl)aminocarbonylmethyloxy-tert-butyl-calix[4]arenes***11c** + **12c** had a total yield of 97% (the ratio of stereomers was 1:1) [19]. After column chromatography (hexane-ethyl acetate, 10:1), the following compounds were isolated:

The first fraction: Calix[4]arene **12c** had a yield of 31%, m.p. 85–87 °C (MeCN). ^1^H NMR (CDCl_3_), δ: 0.55 (t, 3H, Me^Pr^, ^3^*J_HH_* = 6.9 Hz), 0.76 (s, 9H, *t*-Bu), 0.84 (s, 9H, *t*-Bu), 0.87 (t, 3H, Me^Oct^, ^3^*J_HH_* = 7.0 Hz), 1.22-1.32 (m, 12H, -C*H_2_*-^Oct^), 1.33 (s, 9H, *t*-Bu), 1.34 (s, 9H, *t*-Bu), 1.74–1.82 (m, 2H, -C*H_2_*-^Pr^), 1.85 (d, 3H, Me^Amid^, ^3^*J_HH_* = 6.9 Hz), 3.17 (d, 1H, Ar-C*H_2_*-eq, ^2^*J_HH_* = 13.2 Hz), 3.19 (d, 1H, Ar-C*H_2_*-eq, ^2^*J_HH_* = 12.5 Hz), 3.20–3.29 (m, 2H, OC*H_2_*), 3.24 (d, 1H, Ar-C*H_2_*-eq, ^2^*J_HH_* = 12.5 Hz), 3.32 (d, 1H, Ar-C*H_2_*-eq, ^2^*J_HH_* = 13.6 Hz), 3.74–3.93 (m, 2H, OC*H_2_*), 4.18 (d, 1H, Ar-C*H_2_*-ax, ^2^*J_HH_* = 13.6 Hz), 4.21 (d, 2H, Ar-C*H_2_*-ax, ^2^*J_HH_* = 12.5 Hz), 4.24 (d, 1H, Ar-C*H_2_*-ax, ^2^*J_HH_* = 13.2 Hz), 4.44 (d, 1H, C*H_2_*CO, ^2^*J_HH_* = 15.0 Hz), 4.54 (d, 1H, C*H_2_*CO, ^2^*J_HH_* = 15.0 Hz), 5.36–5.43 (m, 1H, C*H*), 6.34 (s, 1H, O*H*), 6.44 (s, 1H, Ar*H*), 6.50 (s, 1H, Ar*H*), 6.54 (s, 1H, Ar*H*), 6.63 (s, 1H, Ar*H*), 7.08 (s, 1H, Ar*H*), 7.09 (s, 1H, Ar*H*), 7.15 (s, 1H, Ar*H*), 7,17 (s, 1H, Ar*H*), 7.25 (t, 1H, Ph*H*, ^3^*J_HH_* = 7.4 Hz), 7.33 (t, 2H, Ph*H*, ^3^*J_HH_* = 7.4 Hz), 7.60 (d, 2H, Ph*H*, ^3^*J_HH_* = 7.4 Hz), 8.77 (s, 1H, N*H*). The calc. % was C 80.95, H 9.30, N 1.45. C_65_H_89_NO_5_. The found % was C 80.37, H 9.53, N 1.06.

The second fraction: Calix[4]arene **11c** had a yield of 33%. The structure and spectral characteristics coincide with compound **8b**.

*Propyloxy-octyloxy-N-(α-phenylethyl)aminocarbonylmethyloxy-tert-butyl-calix[4]arenes***11d** + **12d** had a total yield of 98% (*de* of 20%) [19]. After column chromatography (hexane-ethyl acetate, 10:1), the following compounds were isolated:

The first fraction: Calix[4]arene **12d** had a yield of 28%, m.p. 71–74 °C (MeCN). ^1^H NMR (CDCl_3_), δ: 0.55 (t, 3H, Me^Pr^, ^3^*J_HH_* = 7.4 Hz), 0.82 (s, 9H, *t*-Bu), 0.87 (s, 9H, *t*-Bu + 3H, Me^Oct^, 1.22–1.44 (m, 10H, -C*H_2_*-^Oct^), 1.29 (s, 9H, *t*-Bu), 1.33 (s, 9H, *t*-Bu), 1.53 (d, 3H, Me^Amid^, ^3^*J_HH_* = 6.9 Hz), 1.75–1.93 (m, 2H, -C*H_2_*-^Oct^ + 2H, -C*H_2_*-^Pr^), 3.17 (d, 2H, Ar-C*H_2_*-eq, ^2^*J_HH_* = 12.6 Hz), 3.22 (d, 1H, Ar-C*H_2_*-eq, ^2^*J_HH_* = 13.4 Hz), 3.37 (d, 1H, Ar-C*H_2_*-eq, ^2^*J_HH_* = 13.3 Hz), 3.71–3.93 (m, 4H, OC*H_2_*), 4.08 (d, 1H, C*H_2_*CO, ^2^*J_HH_* = 15.8 Hz), 4.19 (d, 1H, Ar-C*H_2_*-ax, ^2^*J_HH_* = 13.4 Hz), 4.30 (d, 1H, Ar-C*H_2_*-ax, ^2^*J_HH_* = 12.6 Hz), 4.32 (d, 1H, Ar-C*H_2_*-ax, ^2^*J_HH_* = 13.3 Hz), 4.33 (d, 1H, Ar-C*H_2_*-ax, ^2^*J_HH_* = 12.6 Hz), 4.69 (d, 1H, C*H_2_*CO, ^2^*J_HH_* = 15.8 Hz), 5.22–5.30 (m, 1H, C*H*), 5.63 (s, 1H, O*H*), 6.53 (s, 1H, Ar*H*), 6.56 (s, 1H, Ar*H*), 6.62 (s, 1H, Ar*H*), 6.65 (s, 1H, Ar*H*), 7.08 (s, 1H, Ar*H*), 7.09 (s, 2H, Ar*H*), 7,10 (s, 1H, Ar*H*), 7.27 (t, 1H, Ph*H*, ^3^*J_HH_* = 7.0 Hz), 7.37 (t, 2H, Ph*H*, ^3^*J_HH_* = 7.0 Hz), 7.48 (d, 2H, Ph*H*, ^3^*J_HH_* = 7.0 Hz), 8.61 (d, 1H, N*H*, ^3^*J_HH_* = 7.5 Hz). The calc. % was C 80.95, H 9.30, N 1.45. C_65_H_89_NO_5_. The found % was C 79.37, H 8.93, N 1.56.

The second fraction: Calix[4]arene **11d** had a yield of 47%. The structure and spectral characteristics coincide with compound **9b**.

## 4. Conclusions

As a result of this research, it has been shown that anhydrous barium hydroxide is a very convenient base for iteroselective [23] alkylation of the third hydroxyl group with both alkyl bromides and bromoacetic acid derivatives (esters and amides). A preparative method of such alkylation has been developed and its possibilities for the design of inherently chiral derivatives are shown herein.

## Data Availability

No new data were created or analyzed in this study. Data sharing is not applicable to this article.

## Sample Availability

Samples of the compounds are available from the authors.
